# Supplementary material for: A Bounded Measure for Estimating the Benefit of Visualization (Part II): Case Studies and Empirical Evaluation
Source: Entropy (Basel). 2022 Feb 16;24(2):282. doi: 10.3390/e24020282 (PMC8871169; doi:10.3390/e24020282)
Supplement: Supplementary file 1 [file entropy-24-00282-s001.zip › entropy-1511763-supplementary.pdf]

Introduction

This questionnaire is part of an investigation into how different knowledge may be used in viewing volume visualization images that were generated using different datasets and techniques. The descriptions of the relevant scanning and rendering techniques are purposely kept brief in the questionnaire. This is to allow participants to use their existing knowledge to answer the questions.

Question: The image on the right depicts a computed tomography dataset (carp). A ray casting algorithm generated the image of three fish using the dataset. Consider the information that the dataset may contain. Which of the following statement about the **actual dataset** is most likely to be correct?

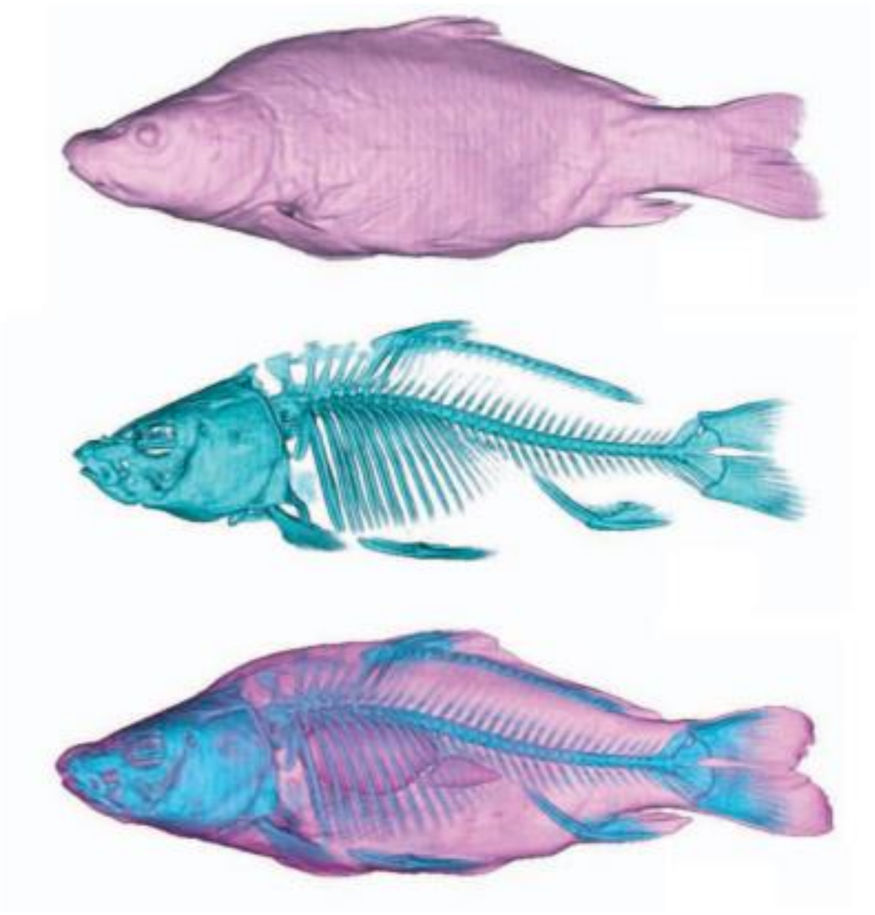

Image by Yingcai Wu and Huamin Qu, 2007

A. The dataset contains three fish, one full fish with pink skin, one fish skeleton with cyan bones, and one half fish with its skeleton laid above.

B. The dataset contains three fish, one full fish, one fish skeleton, and one half fish with its skeleton laid above. It does not contain color information.

C. The dataset contains one fish that has pink skin and cyan bones. The three fish in the image was rendered separately using the same dataset.

D. The dataset contains one fish and does not have any color information. The three fish was rendered separately using the same dataset.

Question: The image on the right depicts a computed tomography dataset (engine block) that was rendered using a ray casting algorithm. Consider the materials that the engine was made of. Which is the most appropriate statement among the following explanations of the **real engine** when it was being scanned?

(A) The engine was partly made of glasses and partly of some red materials. The data was captured and rendered faithfully according to the actual engine.

(B) When the engine was scanned, it consisted only the components in red. The translucent part were added by an artist using some software.

(C) The engine was made of at least two types of materials that are opaque. An algorithm rendered some materials translucent and some in red.

(D) The engine was made of a single type of material that is opaque. An algorithm rendered some part of the object translucent and some part in red.

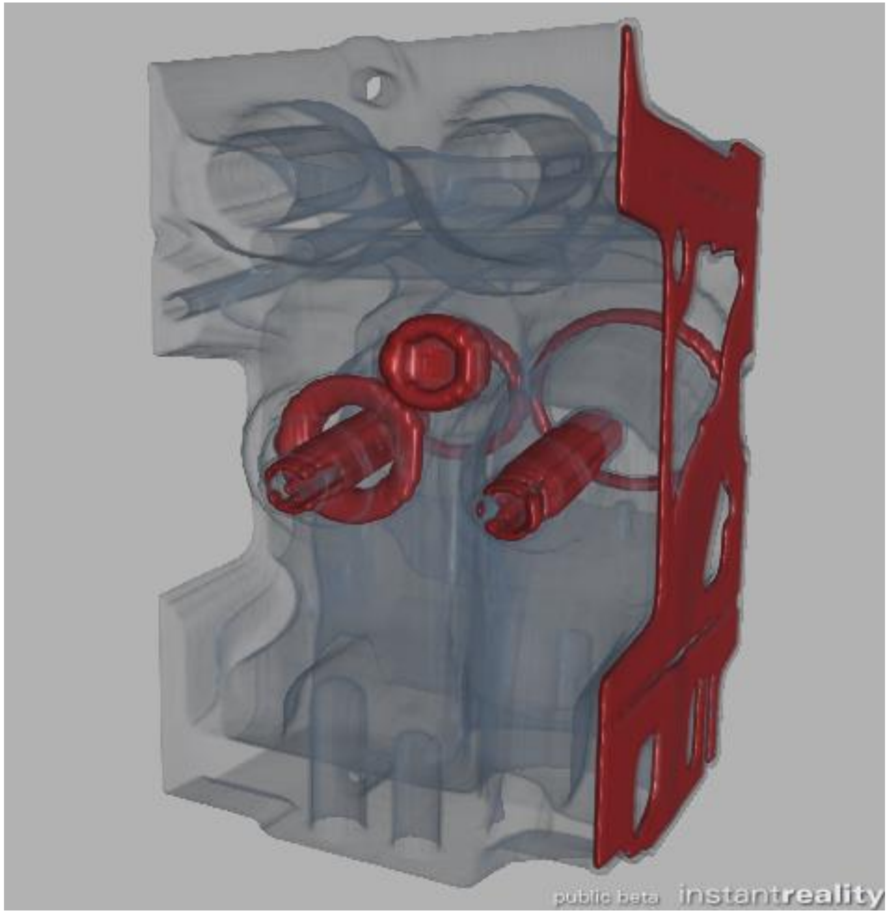

Image by Yvonne Jung, 2008

Question: The image on the right depicts a computed tomography dataset (head) that was rendered using a ray casting algorithm. Consider the section of the image inside the orange circle. Which of the following illustrations would be the closest to the **real cross section** of this part of the facial structure?

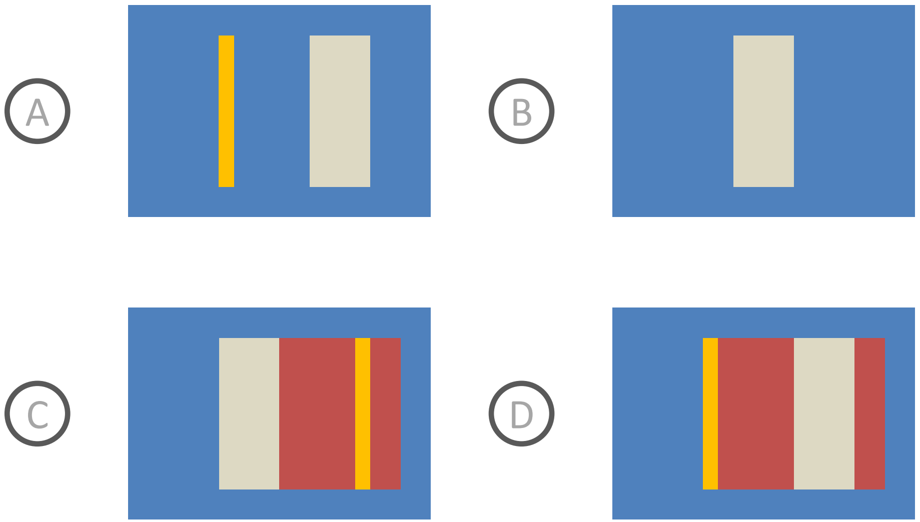

background *bone* *skin* *soft tissue and muscle*

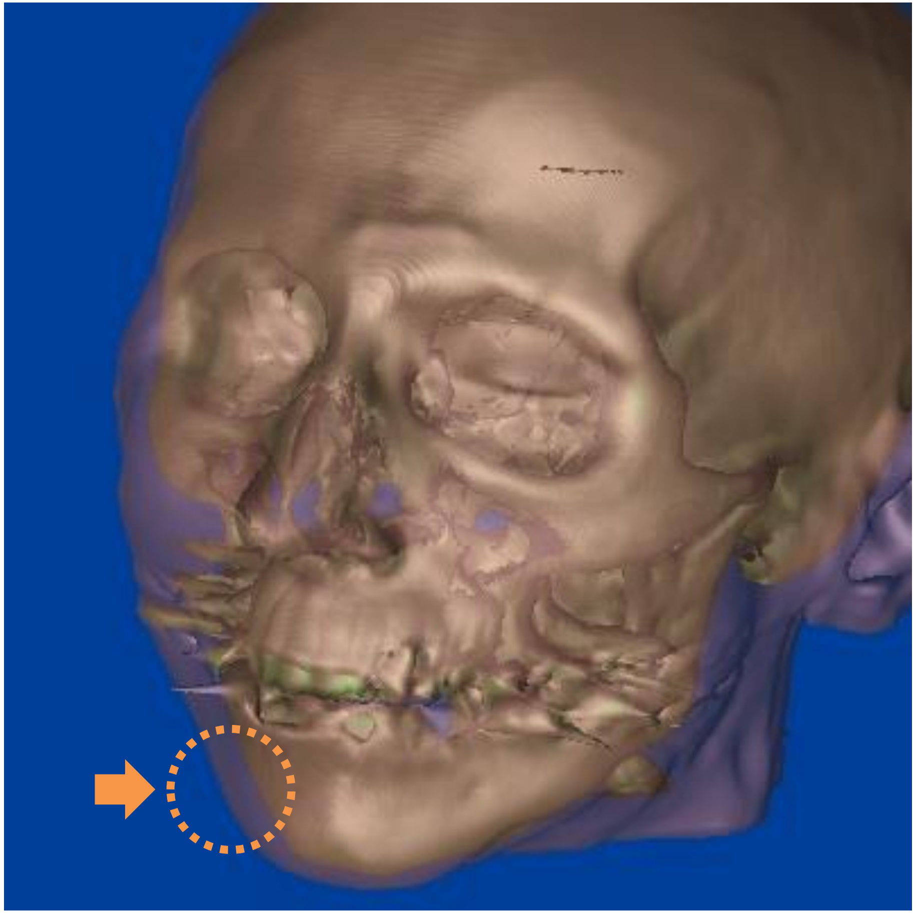

Image by Min Chen, 1999

Question: The image on the right depicts a computed tomography dataset (head) that was rendered using a ray casting algorithm. Consider the section of the image inside the orange circle. Which of the following explanations of the sharp object would be the closest to the **real surface** of this part of the facial structure?

A. The patient has normal teeth. The data was NOT captured correctly. The rendering algorithm correctly revealed the errors in the data.

B. The patient has normal teeth. The data was captured correctly. But an error in the rendering algorithm caused the artefact.

C. The patient has a metal crown. The data was captured correctly. The rendering algorithm correctly shows the effect of a metal object.

D. The patient has a sharp object stuck between his/her teeth. The data and its rendering were both correct.

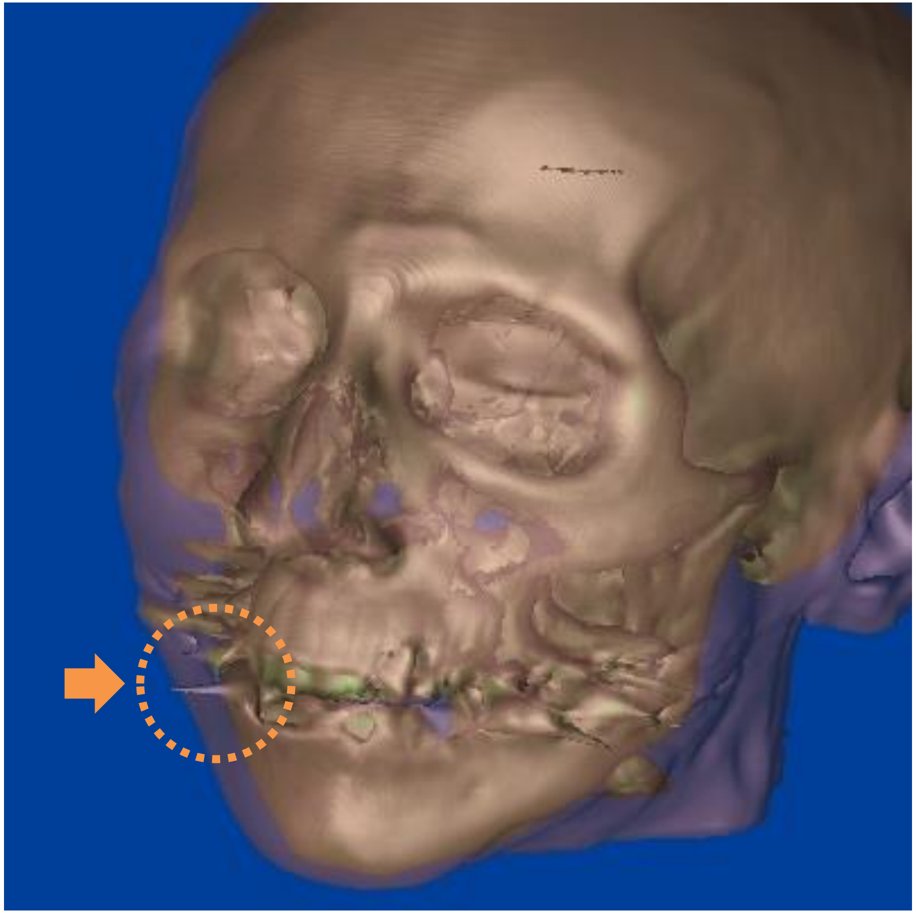

Image by Min Chen, 1999

Question: The image on the right depicts a computed tomography dataset (arteries) that was rendered using a maximum intensity projection (MIP) algorithm. Consider the section of the image inside the **red circle** (also in the inset of a zoomed-in view). Which of the following illustrations would be the closest to the **real surface** of this part of the artery?

A

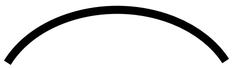

Curved,
rather smooth

B

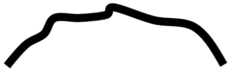

Curved,
with wrinkles and bumps

C

Flat,
rather smooth

D

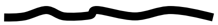

Flat,
with wrinkles and bumps

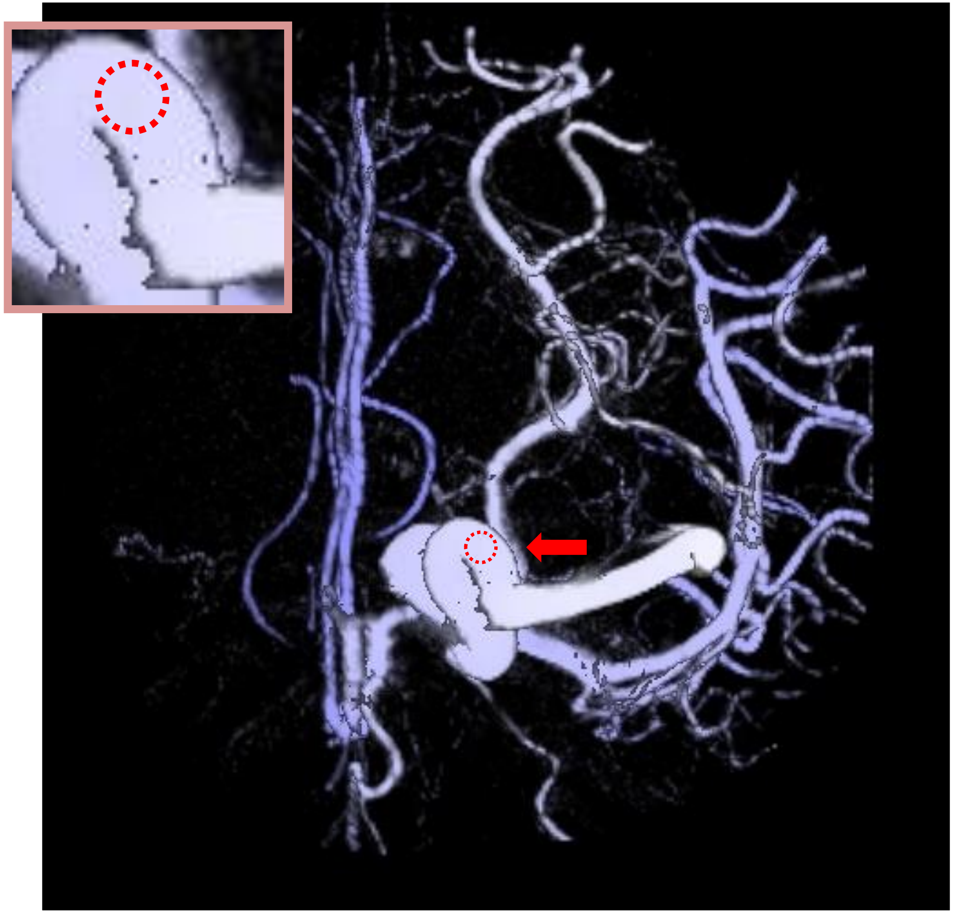

Image by Min Chen, 2008

Question: The image on the right depicts a computed tomography dataset (head) that was rendered using a ray casting algorithm. Consider the part of the image indicated by the **red arrow**. Which of the following explanations of the opening skull in the image would be the closest to the **real head** when it was scanned?

A. The real head was intact. The data captured does not feature the opened skull. A visualization algorithm deformed the skull digitally.

B. The real head was intact. The rendered image originally does not feature the opened skull. An artist modified the image to show the opening.

C. The skull was slightly separated before being scanned. The data was then captured. An algorithm made the opening more dramatic.

D. The skull was fully opened when it was being scanned. The data was captured and rendered faithfully according to the opened skull.

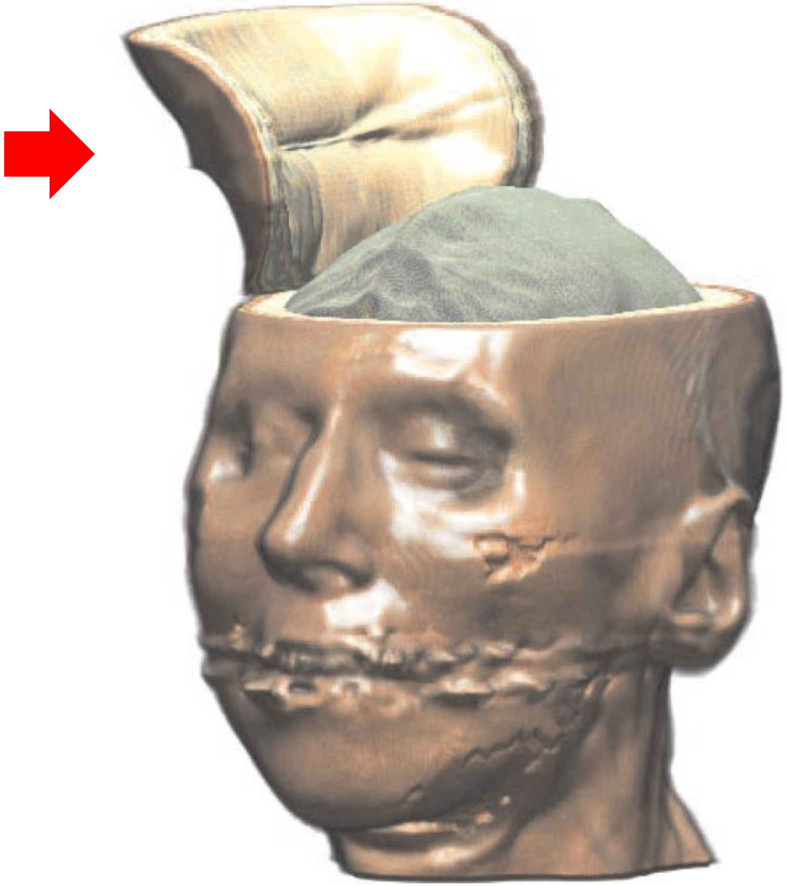

Image by Carlos D. Correa, Deborah Silver and Min Chen, 2006

Question: The image on the right depicts a computed tomography dataset (foot) that was rendered using a non-photorealistic volume rendering algorithm. Consider the part of the image inside the **red circle**. Which of the following statements would be the closest to what you believe to be true?

A. The three toes did not have toe nails when the foot was scanned. This is why the toe nails are apparent in the image.

B. The three toes had toe nails when the foot was scanned. The scanner could not distinguish nails from skins and soft tissues in the captured data.

C. The three toes had toe nails that were captured in the data. The rendering algorithm was not able to show them clearly.

D. The three toes had toe nails that were captured in the data. The nails were removed before the rendering algorithm was applied to the data.

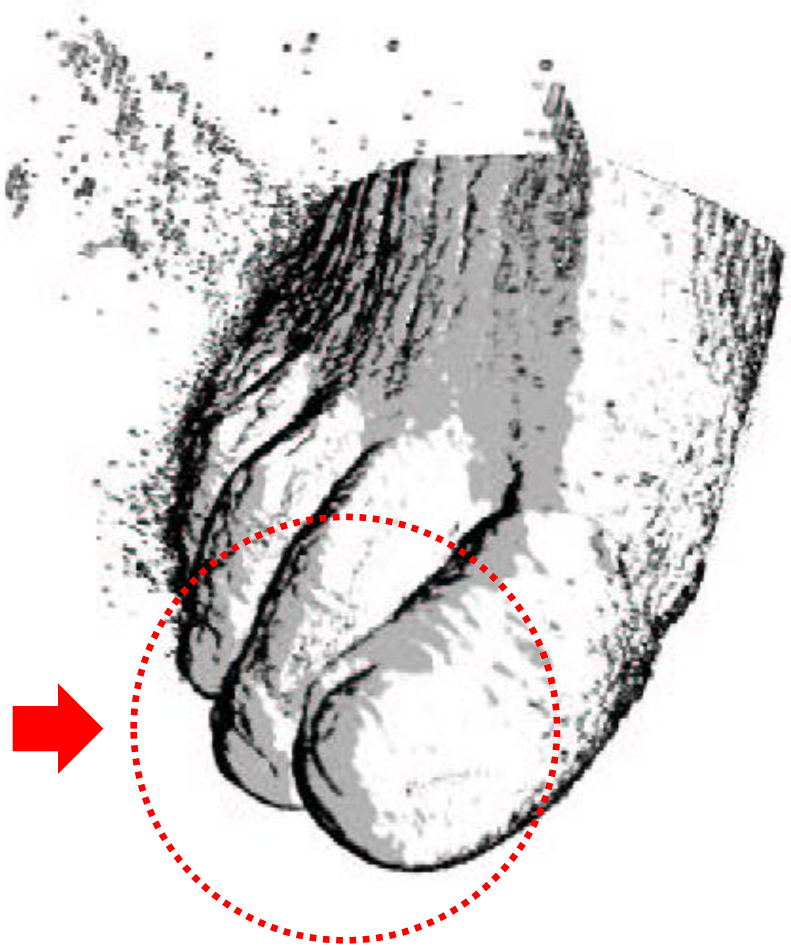

Image by Zoltán Nagy, Jens Schneider and Rüdiger Westermann, 2007

Question: The image on the right depicts a computed tomography dataset (foot) that was rendered using a non-photorealistic volume rendering algorithm. Consider the part of the image inside the orange circle. Which of the following statements would be the closest to what you believe to be true?

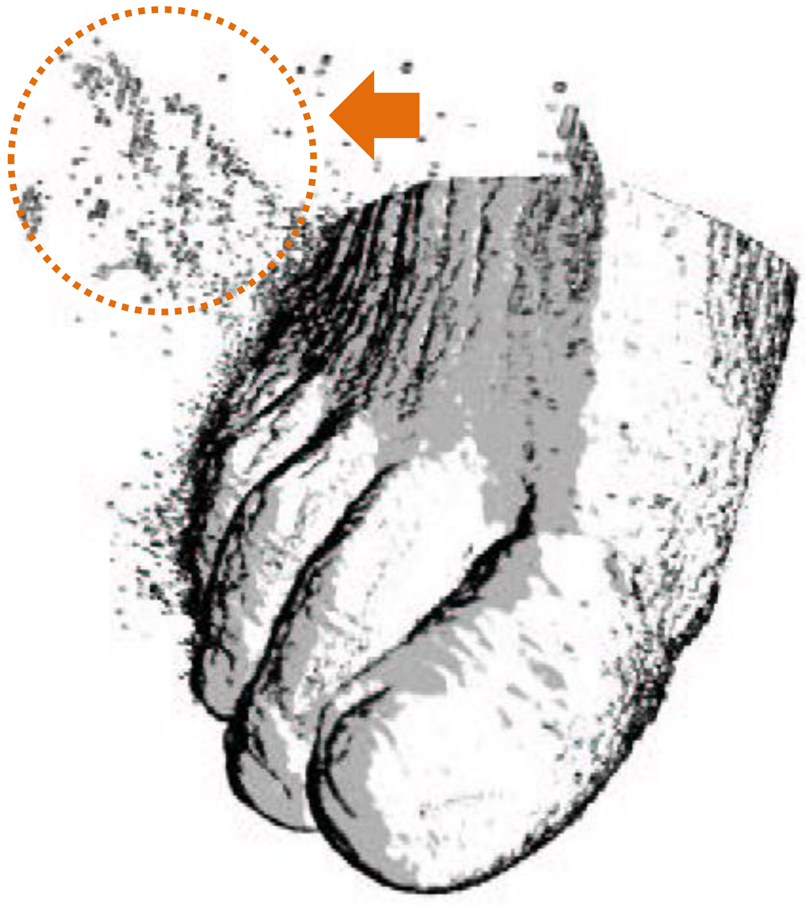

(A) These particles were not part of the dataset. They were introduced as artistic effects by the artist who created this illustrative visualization.

(B) These particles correspond to some kind of noise in the dataset. The rendering algorithm was not able to remove such noise.

(C) These particles correspond to some kind of meaningful signal in the dataset. They were purposely depicted to reveal such information.

(D) These particles were tissues that became disintegrated when the foot was being scanned. They were captured and rendered faithfully.

Image by Zoltán Nagy, Jens Schneider and Rüdiger Westermann, 2007

Please rate your knowledge about 3D medical imaging technology

1. I never heard about 3D medical imaging before
2. I heard about 3D medical imaging before.
3. I saw such devices working and the resulting imagery data before
4. I am not an expert but have worked with such devices or data
5. I am an expert of medical imaging

Please rate your knowledge about volume rendering techniques

1. I never heard about volume visualization or volume rendering before
2. I heard about volume visualization (or volume rendering) before
3. I saw such techniques working and the resulting visualization images before
4. I am not an expert but have worked with volume data and used volume rendering software
5. I am an expert of volume visualization

Thank you very much for answering the questions in this questionnaire
